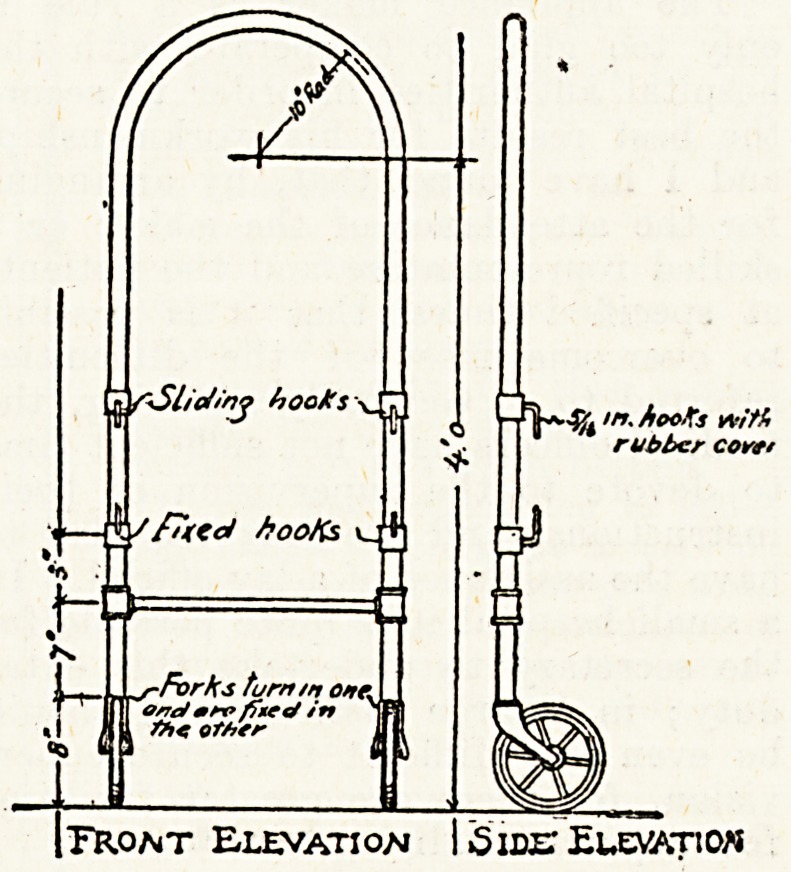# The Institutional Worker

**Published:** 1913-05-31

**Authors:** 


### THE INSTITUTIONAL WORKER

**Being a Special Supplement to "The Hospital"**

### OUR BUREAU OF INFORMATION.

### Rules for Correspondents.

1. Every letter must be accompanied by the coupon to be cut from the back cover (inside page) of The Hospital, current issue, and must contain the name and address of the correspondent with pseudonym for publication if desired. Replies by post cannot be given, save under exceptional circumstances at the Editor's discretion.

2. Letters from Approved Homes in reply to special needs published in the Bureau should state terms and full particulars, and be sent prepaid under cover to the Editor of the Bureau with name written across coupon for identification.

3. Proprietors of Homes which have not yet been entered on the List of Approved Homes, but have spare accommodation likely to suit special needs, are invited to write for an application form for registration. The fee for registration, which includes two announcements of the Home in the Bureau and other privileges, is 10s.

4. All communications to be addressed to the Editor of The Hospital, 28 Southampton Street, Strand, London, W.C., and marked "Bureau of Information."

### *THE SICK AND DISTRESSED.*

**The Editor is prepared to make known without charge the needs of any who may be sick or in difficulties, and to guide them in making choice of Homes for treatment or convalescence. Reduced terms can often be arranged with Homes on the Approved List for those unable to pay full fees. Queries are specially invited from those who are engaged in any kind of philanthropical work. A new edition of the leaflet describing the purposes of the Bureau of Information is now ready and will be sent free of charge on application.**

### After-care of Mental Cases.

The after-care of those discharged from the hospitals and infirmaries is recognised, in an increasing degree, as a necessary adjunct of institutional treatment, for without such care much of the benefit derived from the most skilled surgical and medical treatment may be negatived. Yet, notwithstanding the want, this important branch of charitable aid is in its infancy. A class of patients who are perhaps in greater need of after-care than any other are those mentally afflicted persons who are discharged recovered from the mental hospitals, and the strongest evidence of this is to be found in the fact that the prevention of relapse can only in many instances be secured by rendering the patients such assistance as will relieve them from undue anxiety. The After-Care Association for Poor Persons, discharged recovered from asylums for the insane, is doing much useful work in this direction. This is the only charity of the kind in the United Kingdom, and cases are assisted from all parts of the country, though unfortunately its funds are too limited to do more than reach the fringe of the need. The most important function of the Association is finding suitable occupation for persons who, although recovered, have often many peculiarities and prejudices; and much personal care and individual attention is bestowed upon each case by the workers of the Association, which in itself is even more efficacious than the tangible assistance which is given as well. The offices of the Society are at Church House, Dean's Yard, Westminster, S.W.

### Approved Home.

Roydon, Essex. Tyler's Cross Farm. Principal: Miss Hewitt. A Home for medical, surgical, and maternity cases, and delicate children. Special features: Rest-cure, Weir-Mitchell treatment, and massage. Terms from two guineas weekly.

### *THE INSTITUTIONAL OFFICER.*

### Provision of Surgical Appliances.

A Provincial Secretary writes: I have read the article which appeared in last week's Hospital with much interest; the need for reform in the method of supply of surgical appliances to patients is borne out by my experience. I am convinced, from my own observations, that the plan of sending the patient to the appliance maker is a bad one, for it renders control by the hospital authorities almost impossible; and, moreover, if the patients are permitted to receive their appliances direct from the makers, it depends entirely upon the good sense of the former as to whether they present themselves at the hospital for subsequent examination.

Co-operation with Appliance Maker.

The appliance maker as a rule is only too glad to co-operate with the hospital authorities in order to secure the best results for his workmanship, and I have found that, by arranging for the attendance of the maker or a skilled representative and the patients at specified times, that it is possible to overcome most of the difficulties referred to. Generally speaking, the medical officers have not sufficient time to devote to the supervision of their instructions, and are only too glad to have the assistance of a lay official. In a small hospital it is quite possible for the secretary to undertake this extra duty; in a large institution it should be even less difficult to secure supervision, for there are greater facilities for departmentalising.

The Plan Adopted.

An arrangement should be made with the medical officers to enter the requirements of the patients in a book, together with any special instructions. The officer dealing with the work can then, at the specified times, discuss the needs of the patient with the appliance maker, leaving it to him to secure the object aimed at in the best possible way, and at the same time make such arrangements as may be necessary for helping the patient from the Samaritan Fund or through other agencies. In dealing with the female patients, the help of a nurse can always be arranged for. The appliances should be sent to the hospital for distribution; for by adopting this plan it is possible to make sure that the patients are examined by the medical officers before their discharge. I have for several years supervised this work upon the lines indicated, and I am confident that such time as I have spent upon it has been quite justified by the efficiency and economy which have been effected. Moreover, the work brings one into closer touch with the needs of the patients, who are almost invariably grateful for the attention shown them.

### Geological Formation.

You can obtain information of a general nature regarding geological formation from the National Geological Museum, Jermyn Street, London, S.W. —"Artesian."

### *INSTITUTIONAL FACTS AND FIGURES.*

**QUESTION FOR MAY.**

**During a period of four weeks in th winter months state what was the quantity of gas consumed in your institution for purposes other than illuminating, its approximate cost, the quantity of each kind of fuel used and its cost, deducting the cost of fuel for laundry purposes (if any).**

**In answering this question the following particulars should be given: (1) Number of wards and size of each (in beds); (2) total number of coal fires (a) in the wards, (b) in the rest of the building; (3) the number of gas-cooking and other stoves and sterilisers; (4) the name of the heating and hot-water supply service.**

**In the ensuing month contributors will be asked to give fuller particulars upon this subject by describing the character of the buildings, apparatus, etc.**

Note.—The figures must be the result of actual observation and computation, not estimates. The best answers will be published, and for each contribution considered by the Editor to be of sufficient interest for publication payment of Five Shillings will be made.

RULES.

The following rules must be observed by all those answering the Facts and Figures Questions:—

1. Answers must be written on one side of the paper. They must bear the name and address of the sender and be accompanied by coupon to be cut from the back cover

(inside page) of the current issue of The Hospital. A pseudonym must be chosen if the name is not to be published.

2. Answers must be addressed to the Editor of The Hospital, 28 & 29 Southampton Street, Strand, London, W.C.; they must be marked in the left-hand corner "Facts and Figures," and must be received before the end of the month.

### Cost of Fuel at Sanatorium.

"Iota" sends the following reply to the question for May:—During a selected four weeks in December the cost of fuel used was as follows:—Six tons of coke, costing £6; 3 tons of coal, costing £2 17s.; ½ ton of railway sleepers for fire-lighting, 10s. There is no gas on the premises. (1) There are 14 wards, two beds in 8 wards, and one each in the remaining six. (2) (*a*) No fires in wards; (*b*) six in the rest of the building. (3) A kitchen range, three furnaces for hot water. One steam steriliser, worked from the boiler which supplies steam for the electric-light plant, the steam laundry, and water-pumping plant; the cost of fuel for the furnace of this boiler is not estimated in the above figures. (4) The building is heated by hot-water radiators from one furnace, and the hot-water supply is Messrs. Dent and Hellyer's installation.

[We should be interested to have details of the electric-light and water-pumping plants, the cost of installation, and annual cost of maintenance of each.—Ed.]

### Water Supply.

A problem which has caused, and still is causing, the managers of many provincial institutions considerable anxiety, is the cost of the water supply; for in some districts this is a very heavy item of institutional expenditure. Generally speaking, the water is charged for by measure; the rate per 1,000 gallons varies in accordance with the quantity consumed, and is not infrequently as high as 1s. 2d. Whilst we do not know of any combined appeals by the institutions making use of the same source of supply for more favourable terms, it is a well-known fact that appeals to the water companies by individual institutions have not met with success.

The Question of Well Sinking.

The possibility of providing an independent source of supply by sinking a well has not, we believe, been overlooked by institutional managers burdened with a heavy water rate; but the trustees of public funds naturally hesitate to incur capital expenditure if there be any doubt as to its fruitfulness. The cost of water to some of the London institutions is less now than formerly, for since the passing of the London Water Act of 1907 the change from the system of charging per measure to that of a fixed rate of 5 per cent. on the assessment has proved in many instances to be advantageous. But even in London institutions deep or artesian wells are resorted to in order to effect a saving on the water bill; and in the hotels they are of frequent incidence. These facts should prove suggestive to provincial managers as indicating the desirability of giving the matter careful consideration.

Rain Water.

The storage of rain water for laundry use is a matter which deserves greater attention than it receives, and doubtless the difficulty experienced in obtaining a clean supply is detrimental to the serious consideration of it. There are, however, several simple automatic devices by which this difficulty may be overcome.

We propose in later issues to deal with both these sources of water supply in a series of descriptive notes, which we hope will prove useful and suggestive. Meanwhile, we should be interested to have the views and experiences of our readers upon the subject.

---

### *THE INSTITUTIONAL ARTIFICER.*

### Bed Trucks.

The need for a simple and inexpensive mechanical contrivance for moving beds for a considerable distance, as into the verandah or the grounds, is much felt at many institutions, for the heavy labour entailed in moving beds without some mechanical aid not infrequently hinders those responsible for the care of the patients from taking advantage of the grounds for giving open-air treatment, as often they otherwise would. The spring weather and warm sunshine suggests to us that this matter is one to which attention might be usefully directed, and as a suggestion to institutional artificers and other officers interested in home-made furniture, we publish a description of a pair of simple bed trucks, together with a sketch, for which we are indebted to an account of some home-made furniture in use at the Massachusetts General Hospital published in Charlotte Aiken's book on hospital management.

The apparatus consists of two trucks made of 1-in. tubing, one with a pair of wheels in fixed forks, the other with a pair of wheels on pivots. The one with fixed wheels is clamped to the lower end of the bed, the other to the head, thus converting the bed into a truck. The bed is steered from the head. It will be noticed from the design that the means of clamping the trucks to the bed is provided by a pair of fixed hooks on the standards of each, upon which the lower rails of the bed rest, and by a pair of sliding hooks which grip the upper rails and keep the trucks in position. Doubtless there are many institutional artificers who have designed similar appliances, the description of which will be useful and interesting to our readers.

### *EMPLOYMENT AND TRAINING.*

### Age Limit for Probationers.

There is no reason why you should not take up nursing as a profession at thirty-two years of age, provided that you are suitable in other respects, though your age does increase the difficulty of obtaining a three-years' training. There are a number of training schools which accept candidates up to thirty-three, thirty-four, and thirty-five years of age, as you will note, if you read carefully your book "How to Become a Nurse"—*e.g.* the Guest Hospital, Dudley, the General Kent and Canterbury Hospital, the Leicester Infirmary, and some of the large Scotch hospitals, where older women are preferred. The fact that there may be a large number of applicants at some of these institutions does not preclude the possibility of your success. Some of the smaller provincial hospitals, where you can obtain quite a good training, have considerable difficulty in filling their vacancies satisfactorily, and you might with advantage reply to the advertisements in *The Nursing Mirror*.—"Disappointed."

### Holiday Engagement.

The suggestion you make is, we believe, impracticable. In any case the payment of an honorarium is a business matter, and you can test its feasibility in the usual way through the ordinary channels.—"B. R."

### Period of Nurse's Training.

A coupon should be enclosed with each inquiry; we cannot give postal replies. It is not customary at the general hospitals to issue certificates for periods of training of less than three years; two years' certificates are, however, issued by some of the Poor-Law infirmaries. As on account of your age you may experience some difficulty in getting the training you desire, you might, if unsuccessful, direct your attention to obtaining a post at a cottage hospital, where, doubtless, you could get all the experience you need. You will find full information upon this subject, together with the conditions upon which each institution receives candidates for training, in the book "How to Become a Nurse," price 2s. 3d. post free, published by The Scientific Press, Ltd., 28 and 29 Southampton Street, Strand, London, W.C.—"G. F."

---

### *EDITOR'S LETTER-BOX.*

Communications have been received and attended to from Miss Eastly and Miss Kerr. The Editor is indebted for testimonial in response to inquiry from Mrs. Geary.

The Editor will be glad to receive correspondence and to consider contributions upon all subjects relating to institutional work, and affecting the welfare of institutional officers.

### VACANT MEDICAL APPOINTMENTS.

### Honorary.

Assistant Surgeon, Royal Victoria Eye and Common, S.W.
Gynæcologist, Bolingbroke Hospital, Wandsworth Ear Hospital, Dublin.

### Medical Superintendents, Resident Medical Officers, and other Appointments.

Anæsthetist (£21), Chelsea Hospital for Women.

Assistant House Surgeon (unmarried) (at rate of £75), Royal Albert Hospital, Devonport.

Assistant Medical Officer (at rate of £100), Bethnal Green Infirmary and Workhouse, Cambridge Heath, E.

Assistant Medical Officer (£125), Birmingham Union, Dudley Road Infirmary.

Assistant Medical Officer (unmarried) (£160), Canterbury Borough Asylum.

House Physician (unmarried) (£100), Cheltenham General Hospital.

House Surgeon (unmarried) (£140), Alnwick Infirmary.

House Surgeon (unmarried) (for six months, at rate of £100), Bridgwater Hospital.

House Surgeon (£100), Cossham Memorial Hospital, Kingswood, Bristol.

House Surgeon (£100), Denbighshire Infirmary, Denbigh.

House Surgeon (unmarried) (£100), Dorset County Hospital, Dorchester.

House Surgeon and Secretary (£150), Aberystwyth Infirmary and Cardiganshire General Hospital.

Junior Assistant Medical Officer (unmarried) (£210), County Mental Hospital, Burntwood, near Lichfield, Staffs.

Junior Assistant Medical Officer (unmarried) (£130), Down District Lunatic Asylum, Downpatrick.

Junior Demonstrator in Anatomy (£150), Birmingham University.

Junior House Surgeon (£80), Birkenhead Borough Hospital.

Junior Resident House Surgeon (£100), North Lonsdale Hospital, Barrow-in-Furness.

Locum Tenens Assistant Medical Officer (for three months) (£4 4s. per week), Brighton County Borough Asylum, Haywards Heath.

Medical Superintendent (£500), North Wales Counties Asylum, Denbigh.

Obstetric and Ophthalmic House Surgeon (for six months) (at the rate of £75), Bristol Royal Infirmary.

Physiological Chemist (£350), Cancer Hospital, Fulham Road, S.W.

Resident Assistant Medical Officer (female) (at rate of £100), Dewsbury Union Workhouse.

Resident Medical Officer (unmarried) (£90), Kent and Canterbury Hospital, Canterbury.

Second Assistant Medical Officer (£225), Worcestershire Asylum, Bromsgrove.

Senior House Surgeon (£120), Chesterfield and North Derbyshire Hospital.

Third House Surgeon (£75), Birmingham and Midland Eye Hospital.

Third House Surgeon (£90), Bolton Infirmary and Dispensary.

### Editor's Notices.

### Contributions:

Contributions should be written, or preferably typed, on one side of the paper only, and all articles sent in are accepted upon the distinct understanding that they are forwarded to The Hospital only.

The Editor cannot undertake to return MSS. not used, but when a stamped directed envelope is enclosed the MSS. may be returned if a special request is made.

Accepted articles and paragraphs of news will be paid for after publication at the scale rate.

### Address:

To prevent delay all contributions and letters on editorial business must be addressed exclusively to the Editor, "The Hospital," 29 Southampton Street, Strand, London, W.C. It is important that this regulation shall be strictly observed.

### Correspondence:

Correspondence on all subjects is invited. The name and address of correspondents must be given as a guarantee of good faith, but not necessarily for publication.

### Special Articles:

Special articles are invited, and questions, inquiries, and paragraphs upon all matters relating to the work, administration, and management of general, special, mental, fever, cottage and convalescent hospitals, sanatoria, homes, institutions, societies and organisations for the treatment or care of the sick, injured and dependants of all classes. Every matter affecting the interests and welfare of the staff of all grades working in these institutions will receive special consideration.

### Administrative Medicine, Research and Items of News:

Special terms will be paid for approved articles on subjects in this wide field by experts who have made some section of it their special study and interest. We wish to give prominence to every movement tending to advance accuracy of diagnosis, efficiency in treatment, and the development of modern methods to eradicate disease and promote the welfare of the suffering.

### A Bureau of Information:

We invite inquiries and applications for information and help in providing for that numerous class of sufferers who require special care or house-room which they cannot obtain in their own homes or secure for themselves. **We cannot, however, prescribe, or recommend practitioners.**

### Books for Review:

Publishers are particularly requested to send advance proofs of any new books of importance whenever possible, as the Editor has made arrangements to publish immediate reviews on a new plan.

### Photographs, Plans, Blocks, and Illustrations:

It is requested that wherever possible MSS. may be accompanied by illustrations in any of the above forms. The name of the sender and of the article to which it belongs should be written on the back of each photograph, drawing, or block for purposes of identification.

### Local Papers:

Newspapers containing reports or news paragraphs should be marked and addressed to the Sub-Editor.

### The Coupon System:

A coupon will be found at the bottom of the third inside page of the cover each week. This coupon must be attached to every question or inquiry to which an answer is desired in The Hospital.

### Manager's Notices.

**Letters relating to the publication, sale, and advertising departments of "The Hospital" must be addressed to The Manager, "The Hospital," The Hospital Building, 28 and 29 Southampton Street, London, W.C.**

**Advertisements:**

To ensure insertion, all advertisements for THE HOSPITAL must reach the Manager not later than Wednesday morning in each week. For scale of charges see pages vi and 5.

**Special Rates** will be quoted for those institutions that agree to send all their vacancy, contract, and public notices to THE HOSPITAL each year, so as to make it a file of such announcements for ready reference.

**Subscriptions** may begin at any time, and are payable in advance. Cheques and Post-Office Orders should be crossed London County and Westminster Bank, Covent Garden Branch, and made payable to the Manager of THE HOSPITAL.

RATES OF SUBSCRIPTION (including Postage).

| *United Kingdom.* | | *Foreign and Colonial.* | |
|---|---|---|---|
| Three Months ... ... | 2s. 0d. | Three Months ... ... | 2s. 6d |
| Six Months ... ... | 4s 0d | Six Months ... ... | 5s. 0d |
| Twelve Months ... ... | 6s 6d | Twelve Months ... ... | 8s. 6d. |

## Figures and Tables

**Figure f1:**